# The ion channel TRPV5 regulates B-cell signaling and activation

**DOI:** 10.3389/fimmu.2024.1386719

**Published:** 2024-04-17

**Authors:** Trisha Mahtani, Hena Sheth, L. K. Smith, Leshawn Benedict, Aurelie Brecier, Nader Ghasemlou, Bebhinn Treanor

**Affiliations:** ^1^ Department of Cell and Systems Biology, University of Toronto, Toronto, ON, Canada; ^2^ Department of Biological Sciences, University of Toronto Scarborough, Toronto, ON, Canada; ^3^ Department of Biomedical and Molecular Sciences, Queen’s University, Kingston, ON, Canada; ^4^ Department of Immunology, University of Toronto, Toronto, ON, Canada

**Keywords:** B cells, signaling, TRPV5, ion channels, transient receptor potential channel

## Abstract

**Introduction:**

B-cell activation triggers the release of endoplasmic reticulum calcium stores through the store-operated calcium entry (SOCE) pathway resulting in calcium influx by calcium release-activated calcium (CRAC) channels on the plasma membrane. B-cell-specific murine knockouts of SOCE do not impact humoral immunity suggesting that alternative channels may be important.

**Methods:**

We identified a member of the calcium-permeable transient receptor potential (TRP) ion channel family, TRPV5, as a candidate channel expressed in B cells by a quantitative polymerase chain reaction (qPCR) screen. To further investigate the role of TRPV5 in B-cell responses, we generated a murine TRPV5 knockout (KO) by CRISPR–Cas9.

**Results:**

We found TRPV5 polarized to B-cell receptor (BCR) clusters upon stimulation in a PI3K–RhoA-dependent manner. TRPV5 KO mice have normal B-cell development and mature B-cell numbers. Surprisingly, calcium influx upon BCR stimulation in primary TRPV5 KO B cells was not impaired; however, differential expression of other calcium-regulating proteins, such as ORAI1, may contribute to a compensatory mechanism for calcium signaling in these cells. We demonstrate that TRPV5 KO B cells have impaired spreading and contraction in response to membrane-bound antigen. Consistent with this, TRPV5 KO B cells have reduced BCR signaling measured through phospho-tyrosine residues. Lastly, we also found that TRPV5 is important for early T-dependent antigen specific responses post-immunization.

**Discussion:**

Thus, our findings identify a role for TRPV5 in BCR signaling and B-cell activation.

## Introduction

B cells are an essential part of the adaptive immune system providing protective immunity through the production of antibodies. This process is initiated through B-cell activation, where the B-cell receptor (BCR) recognizes foreign antigen and triggers signaling cascades through the assembly of a signaling complex with the BCR (1). BCR signaling also produces secondary messengers that contribute to the propagation of signal transduction, including calcium (2). The production of inositol 1,4,5-trisphosphate (IP_3_) through the hydrolysis of phosphatidylinositol 4,5-bisphosphate [PI(4,5)P_2_] via phospholipase C-gamma 2 (PLCγ2) leads to the activation of IP_3_ receptors (IP_3_Rs) on the endoplasmic reticulum (ER) that release calcium stores from the ER into the cytosol (2). The emptying of ER calcium stores triggers the activation of stromal interaction molecule (STIM) proteins on the ER that bind to calcium release-activated calcium (CRAC) channels within close proximity on the plasma membrane. This allows for the influx of extracellular calcium, and this pathway is known as store-operated calcium entry (SOCE) (2). Patients with mutations in SOCE proteins are severely immunodeficient, which demonstrates the importance of these proteins in immune cell activation and function (3).

Previous studies in B-cell-specific knockouts of proteins important for SOCE (IP_3_R1/2/3, STIM1/2) have demonstrated the importance of these proteins in B-cell calcium influx upon activation and B-cell proliferation (4, 5). B cells deficient in any of these proteins are unable to produce interleukin 10 (IL-10) due to poor activation of nuclear factor of activated T cells (NFAT) (4, 5). Surprisingly, despite the impairment in B-cell proliferation, B cells deficient in STIM1/2 mount comparable antibody responses to T-independent (TI) and T-dependent (TD) antigens, while B cells deficient in IP_3_Rs have an acute defect in antibody responses to TD antigens (4, 5). This demonstrates that calcium signaling and SOCE are either dispensable to antibody production or other calcium channels are involved in B-cell calcium signaling upon stimulation.

Transient receptor potential (TRP) ion channels are emerging as key regulators of cell sensing and responses to environmental stimuli. There are 27 members in the human TRP ion channel family, which have a general structure that comprises long intracellular N and C termini, and six transmembrane domains with a pore formed by the fifth and sixth transmembrane domains (6). TRP channels have been found to contribute to immune cell development and function. Deletion of *TRPM7* in mice is embryonically lethal; however, studies on T cell- or B cell-specific knockouts demonstrate the importance of this channel in lymphocyte development and activation (7–11). It has also been shown that TRPV1 is involved in calcium signaling and cytokine production upon CD4^+^ T-cell activation and contributes to pro-inflammatory responses in an EAE model (12). Recently, it was shown that TRPV2 is important for B-cell signaling and immunological synapse formation, as well as B-cell activation and antibody responses to immunization and pathogen challenge (13). Given the calcium-selective nature of TRPV5 and TRPV6 (14), we were interested in exploring the potential role of TRPV ion channels in B-cell biology.

Here, we demonstrate the novel finding that TRPV5 is expressed in B cells and polarizes to the BCR cap upon B-cell stimulation in a BCR signaling-dependent manner. TRPV5 polarization was dependent on PI3K and RhoA signaling. We found that TRPV5 is dispensable for B-cell development in a TRPV5 knockout (KO) mouse model generated by CRISPR–Cas9 targeting. Surprisingly, calcium signaling upon B-cell stimulation was not impacted by loss of TRPV5; however, we demonstrate that TRPV5 KO B cells differentially express other calcium ion channels, which likely compensate for the loss of TRPV5. In contrast, we demonstrate that TRPV5 is important for B-cell activation, as TRPV5 is required for B-cell spreading and antigen aggregation in response to membrane-bound antigen, and B cells deficient in TRPV5 exhibit reduced BCR signal transduction.

## Materials and methods

### Mice

C57BL/6 [wild type (WT)] mice were purchased from Charles River, USA. TRPV5 KO mouse model was generated by TCP (Toronto Centre for Phenogenomics) using CRISPR–Cas9 targeting. Guide RNAs toward regions before exon 4 and after exon 7 of *TRPV5* were used to generate a mutated TRPV5 gene to produce a non-functional or no TRPV5 protein. Heterozygous progeny for *TRPV5* deletion (F1) were backcrossed with WT mice for three generations to generate N1, N2, and N3 heterozygous *TRPV5*-deficient mice. N3 mice were imported to the University of Toronto Scarborough (UTSC) vivarium, and heterozygous breeding was utilized to generate TRPV5 KO mice and WT littermates for experimental use. Mice were used at 2–6 months of age for all functional and biochemical experiments. Mice were housed in a specific pathogen-free animal facility at the University of Toronto Scarborough, Toronto, Canada. All experiments were approved by the Local Animal Care Committee (LACC) at the University of Toronto.

### qPCR screening

For TRPV ion channel screening, splenocytes were isolated from C57BL/6 WT mice, and a single cell suspension was prepared using a 70-µm cell strainer and phosphate-buffered saline (PBS, pH 7.4, Gibco). B cells were purified using negative isolation kit (Miltenyi Biotec Inc. or Stem Cell Technologies) according to the manufacturer’s protocol. RNA from 1 × 10^7^ WT B cells was isolated with Qiagen RNeasy mini kit in combination with RNase-free DNase Kit (Qiagen). RNA concentration and purity were measured using a NanoDrop 1000 (Thermo Scientific), and cDNA was synthesized using qScript cDNA synthesis kit (Quanta Bioscience) in a T100 ThermoCycler (Bio-Rad). One-fifth of the cDNA first-strand reaction was utilized to perform quantitative polymerase chain reaction (qPCR) with PerfeCta SYBR Green FastMix (Quanta Bioscience). Primer sets used for reactions were synthesized by Sigma using sequences from Bertin et al. (12). qPCR reactions were performed in the CFX Connect Real-Time PCR Detection System (Bio-Rad), and relative expression with standard error were calculated from Ct (threshold cycle) values with Microsoft Excel. *GAPDH* was used as a housekeeping gene to determine relative expression of TRPV genes.

For TRPV5 mRNA expression in various B-cell subsets, splenocytes and peritoneal cells were isolated from WT mice. Splenocytes were immunostained with B220–Pacific Blue (PB) (2 μg/ml, Biolegend), IgM–Phycoerythrin (PE) (1 μg/ml, Biolegend), CD93-PE/Cyanine 7 (Cy7) (1 μg/ml, Biolegend), CD19–Peridinin–Chlorophyll–Protein (PerCP) (1 μg/ml, Biolegend), CD23–APC (1 μg/ml, Biolegend), CD21/35-APC/Cy7 (1 μg/ml, Biolegend), and IgD–Brilliant Violet 605 (BV605) (1 μg/ml, Biolegend). Peritoneal cells were immunostained with CD19–PB (2 μg/ml, Biolegend), IgM–PE, CD5–PE/Cy5 (1 μg/ml, Biolegend), and B220–AF647 (1 μg/ml, Biolegend). Specific B-cell subsets were isolated with the FACS Aria II (BD Biosciences), and RNA was isolated from 50 to 500 × 10^3^ cells per subset in Trizol (Thermo). RNA concentration and purity were measured using a DeNovix DX-11 Spectrophotometer (Froggabio), and cDNA was synthesized using EasyScript Plus cDNA synthesis kit (ABM) in a T100 ThermoCycler (Bio-Rad). qPCR reactions were performed with SensiFAST SYBR No-ROX mix (Bioline) in the CFX Connect Real-Time PCR Detection System (Bio-Rad), and relative expression with standard error was calculated from Ct values with Microsoft Excel. *HPRT* was used as a housekeeping gene to determine the expression of TRPV5 in various B-cell subsets. The lowest-expressing population was used to calibrate expression values. Data were analyzed using the non-parametric Mann–Whitney statistical test with GraphPad software.

For TRPV5 and calcium channel/protein expression in WT and TRPV5 KO B cells, B cells were isolated from splenocytes of WT and TRPV5 KO mice using a negative selection kit (EasySep Mouse B cell Isolation Kit, Stem Cell Technologies). RNA from 1 × 10^7^ B cells was isolated with Trizol (Thermo). RNA concentration and purity were measured using a DeNovix DX-11 Spectrophotometer (Froggabio), and cDNA was synthesized using Easy Script Plus cDNA synthesis kit (ABM) in a T100 ThermoCycler (Bio-Rad). qPCR reactions were performed with SensiFAST SYBR No-ROX mix (Bioline) in a Thermo Fisher QuantStudio 3 qPCR system, and relative expression with standard error was calculated from Ct values with Microsoft Excel. *HPRT* was used as a housekeeping gene to determine expression. Data were analyzed using t-test and Holm–Sidak *post-hoc* analysis with Prism GraphPad software.

### Immunoblotting TRPV5

Splenocytes were isolated from C57BL/6 WT mice, and a single-cell suspension was prepared using a 70-µm cell strainer and PBS (pH 7.4, Gibco). B cells were purified using negative isolation kit (Miltenyi Biotec Inc. or Stem Cell Technologies) according to the manufacturer’s protocol. B cells were either unstimulated or stimulated with goat anti-mouse IgM μ chain-specific F(ab′)_2_ (5 μg/ml, Jackson ImmunoResearch Laboratories) when immunoblotting for total TRPV5 levels during B-cell activation. Cells were lysed with Laemmli buffer [2% sodium dodecyl sulfate (SDS), 10% glycerol, 60 mM Tris–Cl (pH 6.8), 0.01% bromophenol blue, and 100 mM dithiotreitol (DTT)]. Lysates were sonicated for 10 s and boiled for 5 min before varying volumes (10–40 µl) were loaded into a 12% SDS-PAGE gel and electrophoresed at 120 V in a Mini-PROTEAN tetra vertical electrophoresis cell (Biorad) for 2 h. Proteins were transferred for 90 min at 110 V in the Mini Trans-Blot Cell (Biorad) onto a polyvinylidene fluoride (PVDF; Millipore) membrane, blocked in 5% skim milk in Tris-buffered saline (TBS) with 0.1% Tween 20 (Bioshop) (TBS-T) for 1 h at room temperature with gentle rocking. Blots were washed 5× for 5 min each with shaking in TBST and immunoblotted overnight at 4°C using rabbit anti-TRPV5 antibody (4ADI, 2 μg/ml) with gentle rocking. Blots were washed and incubated with goat anti-rabbit horseradish peroxidase (HRP) antibody (0.08 μg/ml, Jackson Laboratories) for 1 h with gentle rocking at room temperature. ECL Western Blotting Substrate (Pierce) was used to visualize the bands, and the blot was imaged with Gel-Doc (Bio-Rad).

### Immunostaining B-cell subsets for flow cytometry

Splenocytes, bone marrow, and peritoneal cells were isolated from WT mice. Single-cell suspensions were incubated with various antibodies to distinguish B-cell populations for 1 h at 4°C and washed three times with PBS. Splenocytes were immunostained with B220–PB, CD93–PE/Cy7, CD19–PerCP, CD21/35-APC/Cy7, IgM–PE, IgD–BV605, and CD23–APC as described above. Bone marrow cells were immunostained with B220–PB, IgM–PE, CD24–BV605 (1 μg/ml, Biolegend), and CD43-APC (1 μg/ml, Biolegend). Peritoneal cells were immunostained with CD19–PB, IgM–PE, CD5–PE/Cy5, and B220–AF647. Cells were fixed after washing in 4% paraformaldehyde (PFA) for 10 min at 4°C followed by three washes with PBS. Cells were analyzed using the LSR Fortessa (BD Bioscience) and FlowJo (Tree Star).

For TRPV5 protein expression, cells were permeabilized and washed in permeabilization buffer (0.1% saponin, 3% FBS, and 0.1% sodium azide in PBS). Cells were incubated with rabbit anti-TRPV5 antibody (2.5 μg/ml, Abcam) in fluorescence-activated cell sorting (FACS) buffer (2% FBS, 0.1% sodium azide in PBS) at 4°C for 1 h and then washed three times in permeabilization buffer. Cells were then stained with goat anti-rabbit AF488 (2 μg/ml, Invitrogen) at 4°C for 1 h. Cells were washed three times with perm buffer and analyzed using LSRFortessa and FlowJo. Geometric mean of TRPV5 fluorescence intensity (gMFI) was exported to Microsoft Excel, and data were analyzed with Mann–Whitney test in Prism GraphPad.

### Immunostaining TRPV5 for flow cytometry and confocal microscopy

Splenocytes (5 × 10^6^) were isolated from WT mice, and cells were serum starved in Roswell Park Memorial Institute (RPMI) medium for 10 min and either unstimulated or stimulated for 5, 15, or 30 min at 37°C with goat anti-mouse IgM F(ab′)_2_ (5 μg/ml, Jackson ImmunoResearch Laboratories). Cells were then fixed in 2% PFA for 15 min at 37°C followed by three washes in PBS and permeabilized in 0.1% Triton X-100 in PBS for 5 min at room temperature. Cells were washed with FACS buffer three times. Non-specific binding sites were blocked with 2% BSA, 3% goat serum with Fc block CD16/32 (2 μg/ml, BD Pharmigen) in PBS for 30 min. Cells were washed three times in PBS and incubated with rabbit anti-TRPV5 antibody (5 μg/ml or 4ADI, 10 μg/ml, Abcam) in FACS buffer at 4°C for 1 h followed by three washes in FACS buffer. Secondary antibody staining was completed with goat anti-rabbit Alexa-Fluor (AF) 488 (2 μg/ml, Invitrogen) or with IgM Fab–AF647 (0.75 μg/ml, Jackson ImmunoResearch Laboratories), B220–AF488 (2 μg/ml, Biolegend), and goat anti-rabbit Alexa-Fluor 555 (2 μg/ml, Invitrogen) in FACS buffer. Cells were analyzed on an LSR Fortessa, and data were analyzed with FlowJo software (BD). For confocal microscopy, Z-slice images were acquired with an EM-CCD camera (Hamamatsu) with a ×63/1.4 NA objective on a WaveFX Spinning Disk Confocal Microscope (Quorum Technologies Inc.) with an inverted fluorescence microscope (DMI6000B, Leica). Volocity software (Quorum Technologies Inc.) was used for qualitative analysis and visualization of images.

### Inhibitor treatments

Pharmacological inhibitor concentrations were tested using calcium flux experiments or immunoblotting to determine the concentration where B-cell calcium flux or protein phosphorylation upon crosslinking is abolished (protocol as described below) (15–20). For Blebbistatin, recommended concentrations were utilized (20). Inhibitory concentrations were as follows: PP2 (50 µM, Sigma), BAY61-3606 (20 µM, Millipore), Pictilisib/GDC-0941 (10 µM, Selleck Chem), Rhosin (50 µM, Tocris), and Blebbistatin (50 µM, Selleck Chem).

Splenocytes (5 × 10^6^ cells/ml) for each timepoint were treated for 30 min, except for PP2 treatment (15 min), in RPMI with the inhibitor or equivalent vehicle (DMSO) before stimulation. For Rhosin-inhibitor treatment, cells were stained with anti-IgM prior to permeabilization and shielded from light for the rest of the staining protocol. Cells treated with Syk and Myosin IIa inhibitors (BAY61-3606 and Blebbistatin) were shielded from light upon addition and until fixation. The fixation, permeabilization, staining, and imaging protocol is as mentioned above for TRPV5 immunostaining for confocal microscopy.

### Bilayers

Planar lipid bilayers were prepared by spreading liposomes made with DOPC and 0.0125% biotinylated DOPC in FCS2 chambers (Bioptechs) as previously described (21). Chambers were incubated with blocking buffer (2% FBS, 2 mM MgCl_2_, 0.5 mM CaCl_2_, 1 g/L of D-glucose) after lipid spreading, and the following steps were completed with chamber buffer (0.5% FBS, 2 mM MgCl_2_, 0.5 mM CaCl_2_, 1 g/L of D-glucose). Mono-biotinylated surrogate antigen (40 μg/ml of rat anti-kappa light chain, clone HB58) was tethered to lipids using AF633–streptavidin (2 μg/ml, Invitrogen). Splenocytes isolated from WT or TRPV5 KO mice were pre-incubated at 37°C for 10 min and injected into chambers (1 × 10^7^ cells/chamber) for 1.5 or 10 min and fixed with 2% PFA at 37°C for 15 min. Cells were imaged using an Evolve Delta EMCCD camera (Photometrics) with a HCX PL APO ×100/1.47 NA oil immersion objective on a TIRF microscope (Quorum Technologies) with an inverted fluorescence microscope (DMI6000B, Leica) and to capture interference reflection microscopy (IRM) and fluorescence images. Total area of antigen clusters and antigen intensity were measured using Image J. Data were plotted and analyzed using Prism GraphPad software, and the non-parametric Mann–Whitney test was used to measure statistical significance.

For TRPV5 immunostaining, cells were permeabilized, washed, and blocked. Cells were then stained with anti-TRPV5 antibody (4ADI) for 1 h at room temperature, washed, and incubated with goat anti-rabbit AF488 followed by washing. Z-slice images throughout the cell were acquired with an EM-CCD camera (Hamamatsu) with a ×63/1.4 NA objective with a WaveFX Spinning Disk Confocal Microscope (Quorum Technologies Inc.). Volocity software (Quorum Technologies Inc.) was used for qualitative analysis and visualization of images.

### Cell surface biotinylation

Splenocytes were isolated from C57BL/6 WT mice, and a single-cell suspension was prepared using a 70-µm cell strainer and PBS (pH 7.4, Gibco). B cells were purified using a negative isolation kit (Miltenyi Biotec Inc. or Stem Cell Technologies) according to the manufacturer’s protocol. Splenocytes were either unstimulated or stimulated at a concentration of 15 × 10^6^ cells/ml in PBS for 5, 15, or 30 min at 37°C with goat anti-mouse IgM F(ab′)_2_ (5 μg/ml). Cells were immediately placed on ice after stimulation and washed with 1 ml of ice-cold PBS. Cells were labeled with 2 mM EZLink–NHS–SS–LC–LC–Biotin (ThermoFisher Scientific) for 30 min at 4°C on a rotator. Cells were washed three times in ice-cold PBS with 100 mM glycine to quench excess biotin. Cells were then lysed for 30 min in NP40 lysis buffer [1% NP40, 150 mM NaCl, 200 mM Tris pH 8, 100 mM EDTA pH 8, 100 mM sodium orthovanadate, 100 mM sodium fluoride and a cOmplete Mini EDTA-free protease inhibitor cocktail tablet (Roche)]. Debris was spun out at 15,000 × *g* for 15 min, and the supernatant was moved to a fresh tube. A small aliquot (25 µl) was used to perform a plate-based BCA assay (Pierce) at 37°C for 10 min. Samples were measured using spectrophotometry at 562 nm to determine protein concentration compared to a known protein standard ranging from 25 to 2,500 μg of protein. Equal amounts of protein were incubated with streptavidin-coated silica microspheres (0.22 μm, Bangs Laboratories) overnight at 4°C on a rotator. Leftover lysate was saved as total protein input. Beads were pelleted, and the supernatant was saved as the intracellular protein fraction. The beads were then washed in NP40 lysis buffer three times. Laemmli buffer was added to all lysates: total protein input, unbound protein, and protein bound to beads. Lysates were heated to 95°C for 5 min to allow for release of proteins from the beads and then spun for 5 min at 12,000 × *g*. Samples were run on an 8% SDS-PAGE gel, proteins were transferred to PVDF membranes, blocked in 5% skim milk in TBS-T, washed, and incubated with anti-TRPV5 (0.02 μg/ml, Abcam) and goat anti-IgM (0.4 μg/ml, Jackson ImmunoResearch Laboratories). Donkey anti-goat HRP (0.08 μg/ml, Jackson ImmunoResearch Laboratories) and goat anti-rabbit HRP antibody (0.08 μg/ml, Jackson Laboratories) were used to blot for primary unconjugated antibodies. ECL and SuperSignal West Femto-ECL (Pierce) were used to image the blots on a Gel-doc (Biorad).

### Immunostaining T cell and innate cell subsets

Splenocytes and thymic cells were isolated from WT and TRPV5 KO mice. Spleens were cut into small pieces and incubated in RPMI with collagenase (1 mg/ml, Roche) and DNase (0.1 mg/ml, Sigma) at 37°C with occasional stirring for 20 min to extract various innate immune cells. Single-cell suspensions were obtained and incubated with various antibodies to distinguish cell populations for 1 h at 4°C and washed three times with PBS. Splenocytes and thymic cells were immunostained with CD4–PE (1 µg/ml, Biolegend), CD8–APC (1 µg/ml, Biolegend), and CD3–Fluorescein isothiocyanate (FITC) (2.5 µg/ml, Invitrogen) to compare T-cell populations. Innate immune cell phenotyping of splenocytes consisted of immunostaining with CD11b–PB (2.5 µg/ml, Biolegend), CD11c–APC (1 µg/ml, Biolegend), CD64–PE (1 µg/ml, Biolegend), F4/80-APC/Cy7 (1 µg/ml, Biolegend), MHC Class II–BV605 (1 µg/ml, Biolegend), LY6C–AF700 (2 µg/ml, Biolegend), GR1–FITC (2 µg/ml, Biolegend), and NK1.1–PE/Cy7 (1 µg/ml, Biolegend). Cells were fixed after washing in 4% paraformaldehyde for 10 min at 4°C followed by three washes with PBS. Cells were analyzed on an LSR Fortessa, and data were analyzed using FlowJo (BD).

### Calcium signaling

Primary splenocytes (5 × 10^6^) from WT and TRPV5 KO mice were labeled in HBSS (Hank’s Balanced Saline Solution, Gibco) with 2% FBS and 1 mM Fluo-4 (Invitrogen) to label cytosolic calcium for 30 min at 37°C. This was followed by a quick 5-min labeling step with B220–AF647 (0.25 μg/ml) for 5 min on ice. Cells were washed three times with PBS and resuspended in 450 µl of RPMI with DAPI (1 μg/ml) on ice. Cells were warmed to 37°C for 5 min before taking the basal signal on an LSRFortessa (BD) for 30 s. Cells were then stimulated with anti-IgM F(ab′)_2_ at varying concentrations of 0.5, 5, and 20 μg/ml, and calcium signaling was monitored for 5 min after addition. Extracellular calcium determination was examined through addition of 2 mM EGTA (Sigma) in HBSS (Wisent) with 1% FBS and 1 mM MgCl_2_ prior to warming the cells and recording calcium flux for 2.5 min post BCR stimulation before the addition of 2 mM CaCl_2_. Calcium store measurements were conducted in the same buffer as extracellular calcium measurements and stimulated with 1 mM Thapsigargin (Sigma) before the addition of 2 mM CaCl_2_ 2.5 min post flux. Data were analyzed using FlowJo Software, Microsoft Excel, and Graphpad software.

### Phosphoflow

WT or TRPV5 KO splenocytes (2 × 10^6^) were serum starved for 10 min and either unstimulated or stimulated with anti-IgM F(ab′)_2_ (5 μg/ml) for 5 or 15 min in RPMI. Cells were fixed with 4% PFA for 10 min at 4°C and washed once before permeabilization in 100% ice-cold methanol for 20 min on ice. Cells were washed three times and stained with B220–PB and 4G10–biotin (1:25, Sunnybrook Hospital) for p-Tyr or one of the following at 1:25 (Cell Signalling Technologies): p-CD79a (Y182), p-CD19 (Y513), p-Syk (Y346), p-Akt (S473), p-ERK (T202/Y204), or p-PLCy2 (Y-1217) for 1 h at 4°C. Cells were washed three times and then incubated in Streptavidin AF633 for 4G10–biotin or goat anti-rabbit AF488 for single-molecule phospho-stains for 1 h at 4°C before washing three times. Cells were analyzed using LSRFortessa and FlowJo. Fold change in gMFI of phosphorylated molecules was determined using Microsoft Excel and visualized using Graphpad software. Non-parametric statistical Mann–Whitney test was used to measure statistical significance.

### NFAT

Splenocytes were isolated from WT or TRPV5 KO mice. Splenocytes (5 × 10^6^) were either unstimulated or stimulated with anti-IgM F(ab′)_2_ (5 μg/ml) for 5 or 30 min in RPMI. Cells were fixed with 4% PFA for 10 min at 4°C and washed once before washing in permeabilization buffer (3% FBS, 0.1% saponin, and 0.1% sodium azide). Cells were stained in permeabilization buffer with B220–PB and 1:100 anti-NFAT (Cell Signalling Technologies) for 1 h at 4°C. Cells were washed three times and then incubated with goat anti-rabbit AF488 for 1 h at 4°C before washing three times. Cells were analyzed on an LSR Fortessa, and data analysis was performed using FlowJo. Fold change in nuclear NFAT gMFI (amount of NFAT translocated into nucleus calculated by loss of NFAT signal in cytosol) was determined using Microsoft Excel and visualized using Graphpad software. Non-parametric Mann–Whitney test was used to measure statistical significance.

## Results

### TRPV5 is expressed in B cells

**Figure 1:**
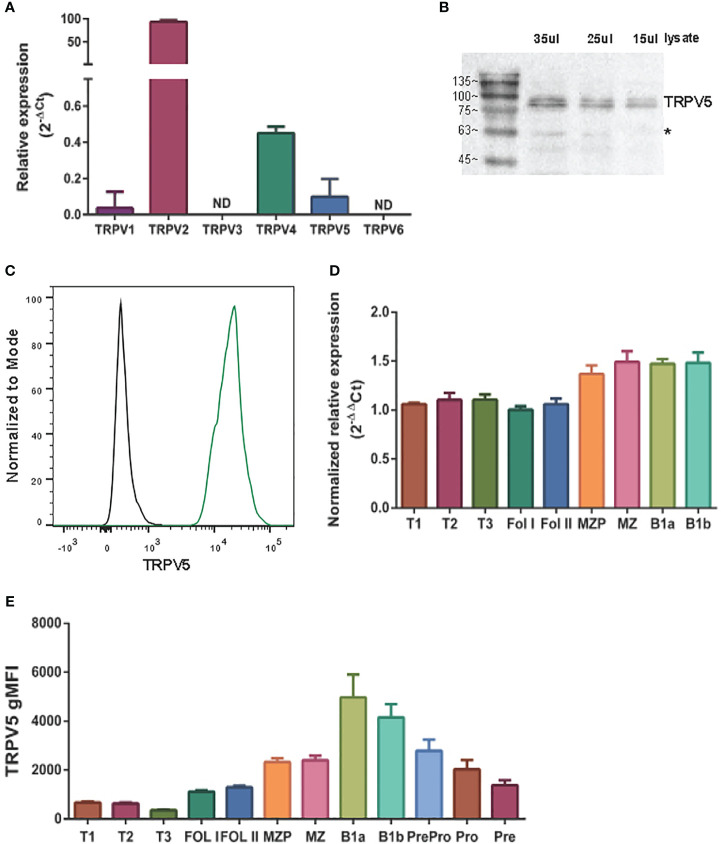
TRPV5 is expressed in B cells. **(A)** RNA isolated from primary murine B cells was synthesized into cDNA for qPCR screening. ΔCt values represent the relative expression of genes normalized to the housekeeping gene *GAPDH*. N.D., not detected. **(B)** Primary murine B cells were lysed, and varying volumes of lysate were subjected to SDS-PAGE followed by immunoblotting with anti-TRPV5 antibody (4ADI). *Non-specific band. **(C)** Primary murine splenocytes were fixed and immunostained for TRPV5 (4ADI) and B220. B-cell expression of TRPV5 was analyzed using flow cytometry through gating on B220^+^ cells stained with anti-TRPV5 antibody (green) or secondary antibody only control (black). **(D)** RNA isolated from FACS-sorted primary murine WT splenic and peritoneal B cells was synthesized into cDNA for a qPCR screen. ΔΔCt values represent the relative expression normalized to the housekeeping gene, *HPRT*, and calibrated to the lowest-expressing FOL I subset. **(E)** Primary splenic, peritoneal, and bone marrow B cells were immunostained for surface markers to identify various B-cell subsets and fixed before immunostaining of TRPV5. Cells were analyzed using flow cytometry. All data are representative of three biological replicates, and error bars represent standard error.

To determine the expression of the TRPV ion channel subfamily in primary murine B cells, we performed a qPCR screen. We found that *TRPV2* and *TRPV4* mRNA was expressed as previously shown in B cells (13, 22); however, we also found an expression of *TRPV5* mRNA (**Figure 1A**). To confirm this new finding, we assessed protein expression using immunoblotting and flow cytometry (**Figures 1B, C**). Immunoblotting of TRPV5 revealed a doublet, as visualized before (23), of the core and glycosylated proteins running between 75 and 100 kDa (**Figure 1B**). We selected TRPV5 as a candidate to study further in B cells, as it is a calcium-selective ion channel (24) that could contribute to B-cell signaling upon stimulation. To investigate the expression of TRPV5 in B-cell subsets, we FACS sorted various B-cell populations in the bone marrow, spleen, and peritoneal cavity (**Supplementary Figure S1**) and performed qPCR to assess the expression of *TRPV5*. We found that *TRPV5* mRNA expression varies between B-cell subsets. Once B cells egress from the bone marrow, they become transitional B cells (T1, T2, T3) before committing to follicular (FOL) or marginal zone (MZ) B-cell fates. Thus, we examined the expression of *TRPV5* in transitional, follicular (FOL I and FOL II), and marginal zone precursors (MZP) and MZ subsets, as well as peritoneal B1 B cells (B1a and B1b). Transitional B cells and FOL B cells had comparable mRNA expression, whereas the innate-like B cells, MZP, MZ, and B1 B cells, have higher mRNA expression of *TRPV5* (**Figure 1D**). These subsets also have higher protein levels of TRPV5 compared to the transitional and FOL B cell subsets, with B1a and B1b cells having the highest levels of TRPV5 (**Figure 1E**). Developing B cells in the bone marrow (Pre-pro, Pro, and Pre B cells) have a stepwise decrease in TRPV5 protein levels, with the lowest level of TRPV5 in Pre B cells, which is comparable to both FOL I and FOL II B cells (**Figure 1E**).

### TRPV5 polarizes to the site of BCR signaling

**Figure 2:**
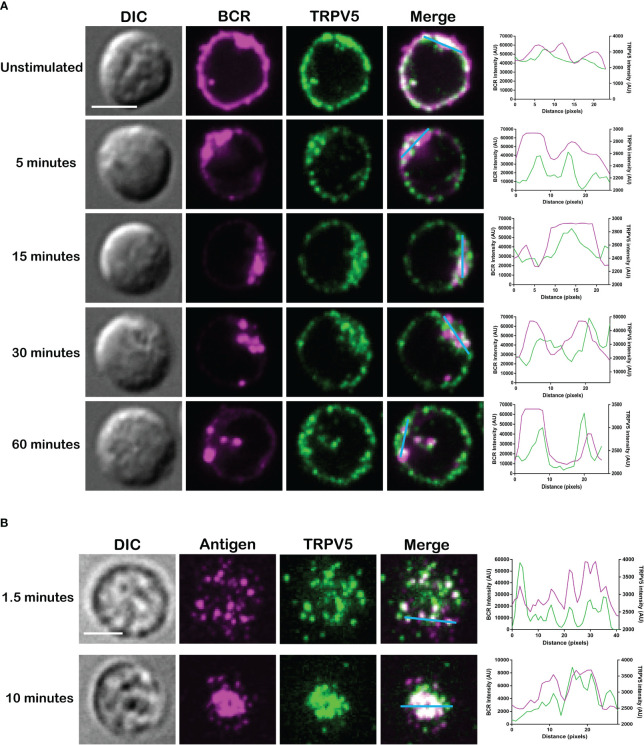
TRPV5 localizes with BCR clusters upon stimulation. Representative confocal fluorescence microscopy images of fixed primary murine splenic B cells immunostained for TRPV5 [green, **(A)** Abcam, **(B)** 4ADI] either **(A)** unstimulated or stimulated for various periods with anti-IgM F(ab′)_2_ and immunostained for BCR (magenta) or **(B)** spread for 1.5 or 10 min on planar lipid bilayers with tethered surrogate antigen, anti-kappa light chain (magenta). Line ROIs (blue) were drawn on the cell using ImageJ. Line profiles were generated using ImageJ for the line ROIs and Plot Profile plugin and were plotted on GraphPad Prism. Scale bar represents 3 µm. Data are representative of two independent experiments and at least 30 cells per condition.

TRPV5 has previously been shown to be localized in intracellular vesicles and at the plasma membrane (25–27). Cell stimuli, such as hormone stimulation, has been shown to increase TRPV5 expression at the plasma membrane through the insertion of vesicle-localized channels into the plasma membrane (25–27). To investigate the localization of TRPV5 in primary murine splenic B cells, we fixed, permeabilized, and immunostained B cells after BCR stimulation to visualize TRPV5 and BCR by confocal microscopy. We found that TRPV5 is predominantly localized at the plasma membrane in unstimulated (steady-state) primary naïve B cells (**Figure 2A**). Upon BCR stimulation with cross-linking pseudo-antigen [anti-IgM F(ab′)_2_], TRPV5 polarized to the site of the BCR cap throughout a 60-min time course (**Figure 2A**). We also observed that TRPV5 localized with BCR in the intracellular puncta from 30 min post-stimulation (**Figure 2A**). B cells typically encounter antigen (Ag) presented on the membrane of antigen-presenting cells (APCs) *in vivo* (28–31) and spread over this presenting membrane to collect as much antigen as possible before Ag : BCR microclusters are aggregated into a central cluster (32–34). Thus, we also probed TRPV5 localization upon membrane-bound antigen stimulation with surrogate antigen tethered to artificial planar lipid bilayers. We observed that TRPV5 localizes with, and adjacent to, BCR microclusters during the B-cell spreading response and localizes with aggregated antigen upon B-cell contraction at 10 min post-contact (**Figure 2B**). These data demonstrate that TRPV5 is recruited to the site of BCR clustering and signaling upon both soluble and membrane-bound antigen stimulation.

### Membrane-localized TRPV5 polarization to BCR clusters is BCR signaling dependent

**Figure 3:**
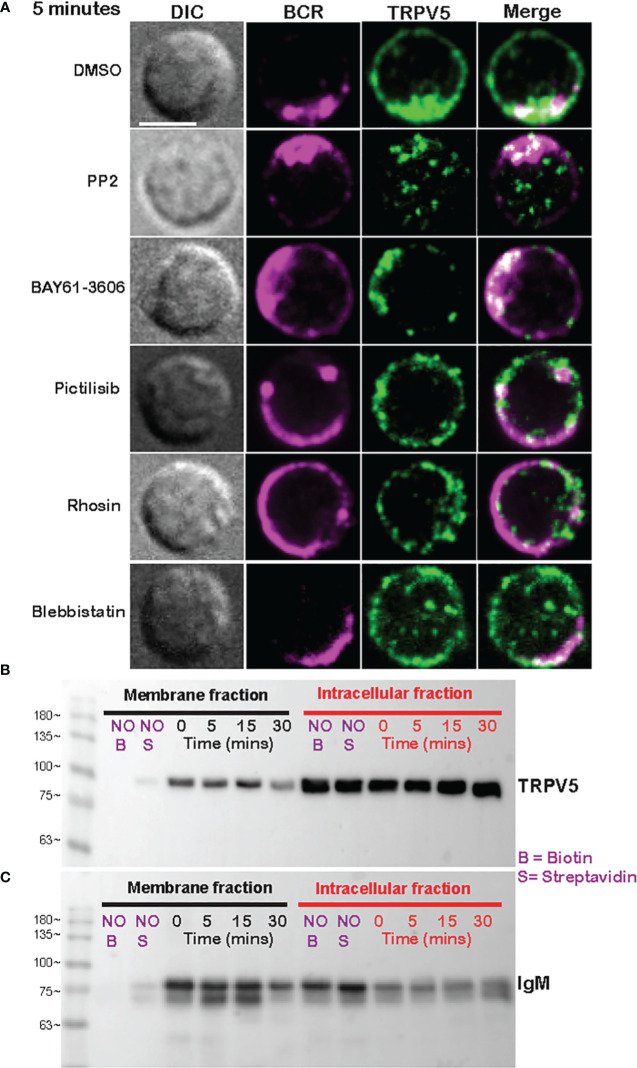
Membrane-localized TRPV5 polarizes to BCR clusters in a signaling-dependent manner. **(A)** Representative confocal fluorescence microscopy images of primary murine splenic B cells treated with either DMSO (vehicle), PP2, BAY61-3606, pictilisib, Rhosin, or Blebbistatin before stimulation for 5 min with anti-IgM F(ab′)_2_. Cells were fixed and immunostained for BCR (magenta) and TRPV5 (green). Scale bar represents 3 µm. Data are representative of two independent experiments and at least 30 cells per condition. **(B, C)** Primary murine splenic B cells were stimulated for various timepoints, and surface proteins were biotinylated. Cells were lysed, and membrane and intracellular protein fractions were separated. Lysates were subjected to SDS-PAGE followed by immunoblotting with **(B)** anti-TRPV5 antibody or **(C)** anti-IgM antibody.

As we see rapid TRPV5 polarization with BCR that is sustained for up to 60 min, we examined whether TRPV5 is recruited to the BCR cap in a BCR signaling-dependent manner using pharmacological inhibitors targeting various kinases involved in B-cell signaling. Inhibition of Src kinases by PP2 did not disrupt BCR capping nor polarization of TRPV5 (**Figure 3A**). Syk kinase inhibition with BAY61-3606 also did not impair TRPV5 polarization upon BCR cap formation (**Figure 3A**). However, we found that inhibition of PI3K by pictilisib did prevent TRPV5 polarization at the BCR cap (**Figure 3A**). To probe how PI3K signaling impairs TRPV5 polarization, we investigated whether inhibition of RhoA impaired TRPV5 polarization. RhoA has been shown to be important for PI(4,5)P_2_ synthesis in B cells (35), and TRPV5 has a strong binding affinity for PI(4,5)P_2,_ which keeps TRPV5 in an active conformation (36, 37). We found that TRPV5 polarization is dependent on both PI3K and RhoA signaling, as inhibition of RhoA with Rhosin also impaired TRPV5 polarization (**Figure 3A**). Since RhoA is also important for activation of Myosin IIa in B cells (38), we examined BCR and TRPV5 polarization in Blebbistatin-treated cells. Consistent with previous findings, inhibition of myosin IIa did not impair BCR clustering; however, it did abrogate TRPV5 polarization in stimulated B cells (**Figure 3A**). As inhibition of myosin impairs BCR : Ag-containing vesicle fusion with MHC Class II-containing vesicles, (38) impairment of TRPV5 polarization in Blebbistatin treated cells may be due to dysregulated vesicle trafficking. Taken together, these findings demonstrate that TRPV5 is recruited to BCR clusters in a signaling-dependent manner and implicate TRPV5 in BCR signaling and activation.

To investigate whether the observed polarization of TRPV5 was due to recruitment of membrane- or vesicle-localized channels, we probed changes in channel localization at the membrane by cell surface biotinylation, as previously shown (39–43). TRPV5 localization at the membrane was mostly unchanged throughout a 15-min time course of antibody cross-linking of BCRs (**Figure 3B**). We did note a decrease in membrane-localized TRPV5 at 30 min post-stimulation consistent with our visual observation of TRPV5 in intracellular puncta that co-localize with BCR at this timepoint. Intracellular TRPV5 levels are also largely consistent during the stimulation time course suggesting that majority of TRPV5 in intracellular compartments is not inserted into the membrane upon BCR stimulation (**Figure 3B**). To verify the efficacy of our protocol to detect changes in cell surface localization, we also examined IgM, which is rapidly internalized upon BCR stimulation. We found that the membrane fraction of IgM decreased during the time course (**Figure 3C**) consistent with previous reports of BCR internalization (44, 45). In fact, the decrease in membrane-localized TRPV5 is similar to the observed internalization of IgM, where the decrease is most noticeable at 30 min post-stimulation. These findings suggest that polarization of TRPV5 upon BCR stimulation is due to the recruitment of membrane-localized channels and not increased trafficking of the channel to the plasma membrane.

### TRPV5 is dispensable for B-cell development

**Figure 4:**
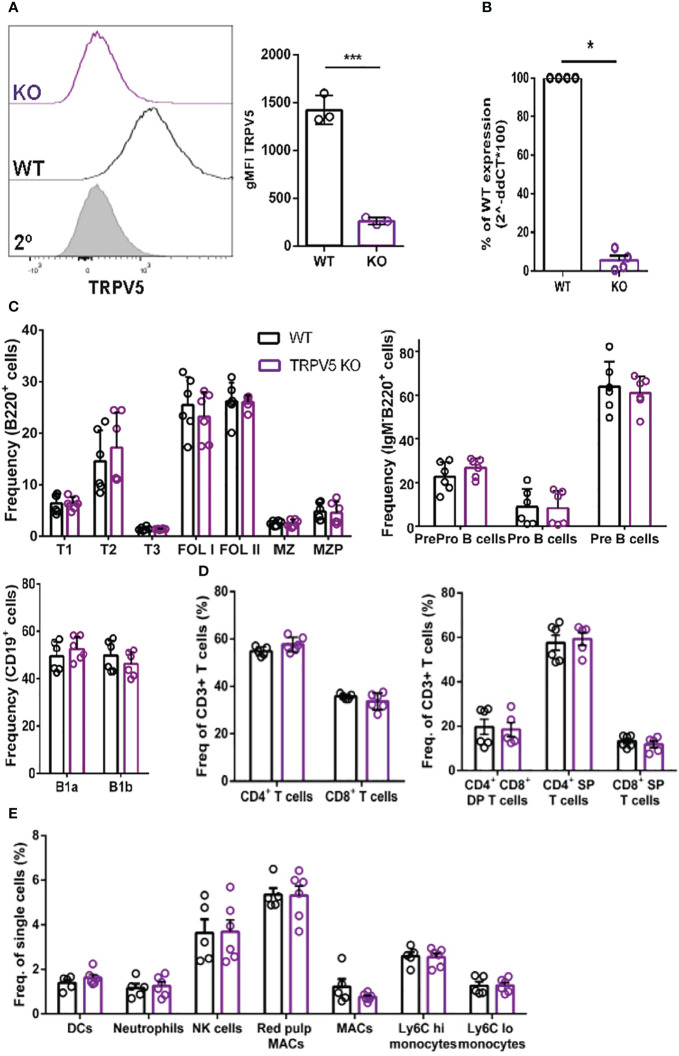
TRPV5 deficiency does not impair B-cell development or mature B-cell subsets. **(A)** Primary C57BL/6 WT and TRPV5 KO murine splenic B cells were fixed and immunostained for TRPV5 and analyzed using flow cytometry; ***p < 0.001. **(B)** For qPCR, splenic B cells were purified from WT and TRPV5 KO mice and lysed in Trizol to extract RNA and synthesize cDNA. Gene expression was measured using qPCR; normalized relative gene expression of TRPV5 KO B cells compared to WT (displayed as 1/ΔCt, normalized expression) was calculated using the ΔΔCt method. *HPRT* was the housekeeping gene for normalization, and error bars represent the standard error; n = 4. Statistical analysis was performed using the Mann–Whitney test; *p = 0.0268. **(C)** Primary bone marrow (right panel), splenic (left panel), and peritoneal (right panel) B cells, **(D)** primary splenic (left panel) and thymic (right panel) T cells, and **(E)** splenic innate immune cells were immunostained for surface markers to identify various subsets and analyzed using flow cytometry. Data are representative of six biological replicates, and error bars represent standard error. Statistical analysis was performed using the Mann–Whitney test.

To probe the role of TRPV5 in B cells, we generated a TRPV5 knockout (KO) mouse model [Toronto Centre for Phenogenomics (TCP)] by CRISPR–Cas9 targeting. CRISPR–Cas9 targeting of the *TRPV5* gene successfully generated a TRPV5 KO as we found no expression of TRPV5 protein (**Figure 4A**) and significantly reduced *TRPV5* mRNA expression in TRPV5 KO B cells in comparison to WT B cells (**Figure 4B**). As we observed variable TRPV5 expression in B-cell subsets, we wanted to determine whether TRPV5 expression was required for B-cell development. We isolated splenocytes, bone marrow, and peritoneal cells from WT and TRPV5 KO mice and immunostained cells for various cell surface markers to gate on specific populations as before (**Supplementary Figure S1**). We found that TRPV5 is dispensable for B-cell development, as developing B cells in the bone marrow were comparable to WT (**Figure 4C**). We also found that loss of TRPV5 expression did not perturb transitional, FOL, or MZ zone B-cell populations in the spleen or B1 B cells in the peritoneal cavity (**Figure 4C**). Therefore, TRPV5 expression is not required for B-cell development. As we generated a global TRPV5 KO mouse model, we also analyzed T-cell and innate immune cell populations using immunostaining and flow cytometry (**Supplementary Figure S2**). We found splenic CD4 and CD8 T-cell numbers in TRPV5 KO mice comparable to WT mice (**Figure 4D**), and further probing into single or double-positive T cells in the thymus also demonstrated no differences (**Figure 4D**). Additionally, we found no differences in total splenic macrophages, red pulp macrophages, DCs, NK cells, neutrophils, or monocytes (**Figure 4E**). Thus, TRPV5 deficiency does not impact lymphocyte, macrophage, DC, monocyte, and neutrophil development or maintenance, as these populations are comparable to WT.

### TRPV5 does not contribute to calcium signaling upon BCR stimulation

**Figure 5:**
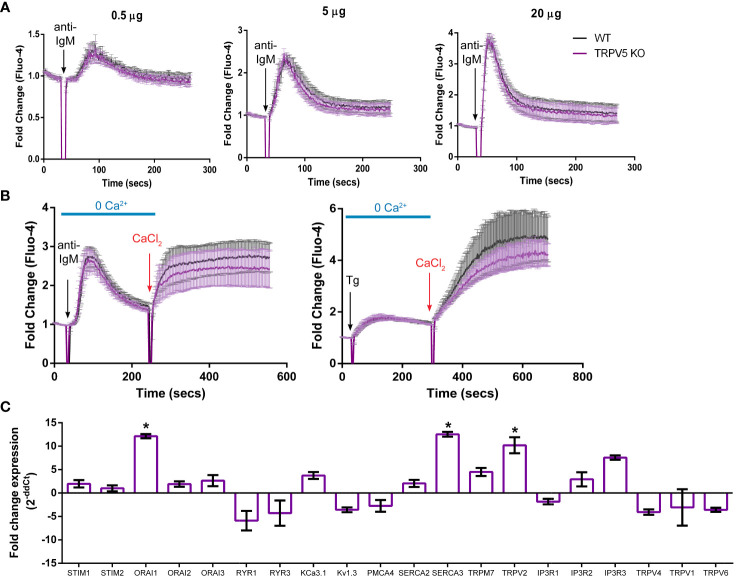
TRPV5 deficiency does not significantly impair calcium signaling upon BCR stimulation. Splenocytes were labeled with the calcium indicator Fluo-4AM and B220-APC. Calcium flux upon BCR stimulation in **(A)** RPMI or **(B)** HBSS, 1% FBS, 1 mM MgCl_2_, and 1 mM EGTA was monitored using flow cytometry. Black arrow indicates addition of **(A, B)** stimulating anti-kappa antibody at varying concentrations or **(B)** 1 mM Thapsigargin. Red arrow indicates the addition of 2 mM CaCl_2_. Fold change of Fluo-4 signal over time in WT (black line) and TRPV5 KO (purple line) B cells. Data are representative of six biological replicates; error bars represent standard error. Statistical analysis on peak calcium and area under the curve (AUC) were performed using Mann–Whitney test. **(C)** Splenic B cells were isolated from WT and TRPV5 KO mice and lysed to extract RNA and synthesize cDNA. Gene expression was measured using qPCR, and normalized relative gene expression of TRPV5 KO B cells compared to WT (displayed as 1/ΔCt normalized expression) was calculated using the ΔΔCt method. *HPRT* was the housekeeping gene for normalization, and error bars represent the standard error. Data are representative of the mean of two technical replicates per biological replicate; n = 4. Statistical analysis was performed using t-test and the Holm–Sidak method. *p<0.05.

To determine the role of TRPV5 in B-cell calcium signaling, we loaded WT and TRPV5 KO B cells with the calcium-specific dye, Fluo-4AM, and monitored calcium signaling kinetics using flow cytometry. Surprisingly, calcium responses in TRPV5 KO B cells were only slightly and non-significantly reduced in comparison to WT B cells at three different F(ab′)_2_ cross-linking antibody concentrations, 0.5, 5, and 20 µg/ml (**Figure 5A**). Calcium elevation post-stimulation is initiated by the emptying of intracellular stores, which is followed by the influx of extracellular calcium. To parse the contribution of intracellular calcium store release from the ER into the cytosol and the extracellular calcium flux mediated by plasma membrane channels, cells can be treated with EGTA [ethylene glycol-bis (β-aminoethyl ether)-N,N,N′,N′-tetraacetic acid] to chelate extracellular calcium, and calcium was added back to the media after measuring intracellular calcium flux. Again, TRPV5 KO B cells exhibited a slight, but non-significant, impairment in both intracellular and extracellular calcium signaling upon BCR stimulation (**Figure 5B**). We further probed whether TRPV5 was important for SOCE by Thapsigargin inhibition of SERCA (sarco/endoplasmic reticulum Ca^2+^-ATPase) channels to bypass BCR stimulation and trigger ER calcium store entry. We found that TRPV5 is dispensable for SOCE signaling in B cells (**Figure 5B**). In comparison, we knocked down the expression of TRPV5 by siRNA knockdown in a murine B-cell line expressing IgM BCR, D1.3 A20 B cells. We found that even modest reduction in TRPV5 expression (~30%) was sufficient to reduce calcium signaling upon BCR stimulation (**Supplementary Figure S3**). This suggests that TRPV5 KO B cells may have compensatory mechanisms that mask any potential differences, as RNAi revealed differences in calcium signaling (**Supplementary Figure S3**). We probed the expression of regulatory proteins and channels known to be involved in calcium responses in murine splenic B cells using qPCR. In comparison to WT B cells, TRPV5 KO B cells had significantly increased mRNA expression of *ORAI1*, *SERCA3*, and *TRPV2* and an overall trend of increased expression of proteins that contribute to or regulate calcium signaling (**Figure 5C**). Thus, TRPV5 KO B cells have compensatory mechanisms in regulating calcium influx and re-filling of stores indicating an important role for TRPV5 in primary murine B cells.

### TRPV5 is necessary for B-cell spreading and contraction response

**Figure 6:**
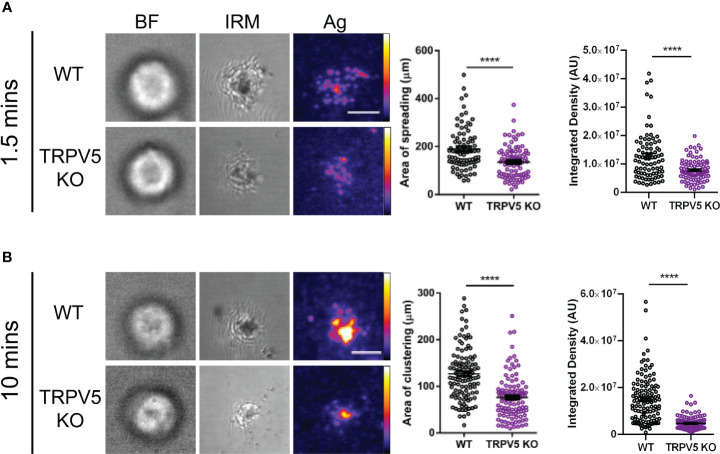
TRPV5 is required for B-cell spreading and antigen aggregation upon stimulation with membrane-bound antigen. Representative total internal reflection fluorescence (TIRF) microscopy images of fixed WT or TRPV5 KO primary murine splenic B cells spread for **(A)** 1.5 or **(B)** 10 min on planar lipid bilayers with tethered surrogate antigen (Ag), anti-kappa light chain. BF, brightfield; IRM, interference reflection microscopy. Scale bar represents 3 μm. Data are representative of four independent experiments and at least 20 cells per condition. Cluster area and density were measured using ImageJ, and graphs represent the mean of four biological replicates; error bars represent standard error. Statistical analysis was performed using the Mann–Whitney test; ****p < 0.0001.

B cells interact with foreign antigen presented on the membrane of APCs *in vivo* forming BCR–antigen microclusters. These microclusters are the initiation site of BCR signaling, and impairment of microcluster formation may be indicative of a signaling defect. As we found that TRPV5 localized to BCR microclusters during membrane-bound antigen stimulation, we wanted to determine whether TRPV5 is necessary for the cell spreading and antigen aggregation process. To investigate this, we allowed WT and TRPV5 KO B cells to settle on artificial planar lipid bilayers with a tethered surface antigen and used both total internal reflection fluorescence (TIRF) microscopy and interference reflection microscopy (IRM) to visualize the interaction. Visual inspection of IRM images revealed a defect in TRPV5 KO B cell contact area, which was mirrored in the TIRF microscopy images that demonstrated a defect in TRPV5 KO B-cell spreading and antigen microcluster formation at 1.5 min. To quantify this observation, we measured the area of cell spreading and the total fluorescence intensity of antigen within this region. We found that at 1.5 min post-stimulation, the area of cell spreading is decreased in TRPV5 KO B cells (135.8 ± 7.6 µm, 71% of WT), and the total intensity of antigen within this region is also reduced [7.9 × 10^6^ ± 4.3 × 10^5^ arbitrary units (AU), 61% of WT] in comparison to WT B cells (area: 190.9 ± 10.0 µm; intensity: 1.3 × 10^7^ ± 9.4 × 10^5^ AU) (**Figure 6A**). We also found that loss of TRPV5 impacted antigen aggregation in B cells, as the area of aggregated antigen at 10 min post-stimulation in TRPV5 KO B cells was 43% smaller (76.2 ± 4.3 µm) and 69% lower in antigen intensity (4.8 × 10^6^ ± 2.6 × 10^5^ AU) in comparison to WT B cells (area: 132.4 ± 7.9 µm; intensity: 1.5 × 10^7^ ± 9.5 × 10^5^ AU) (**Figure 6B**). These findings demonstrate the importance of TRPV5 in B-cell responses to membrane-bound antigen, as TRPV5 KO B cells have a significant reduction in cell spreading, microcluster formation, and a resulting decrease in antigen collection during the contraction response to membrane-bound antigen. As BCR:antigen microcluster formation leads to the formation of signaling microsignalosomes, these data suggest that loss of TRPV5 may impact BCR signaling.

### TRPV5 is important for BCR signal transduction

**Figure 7:**
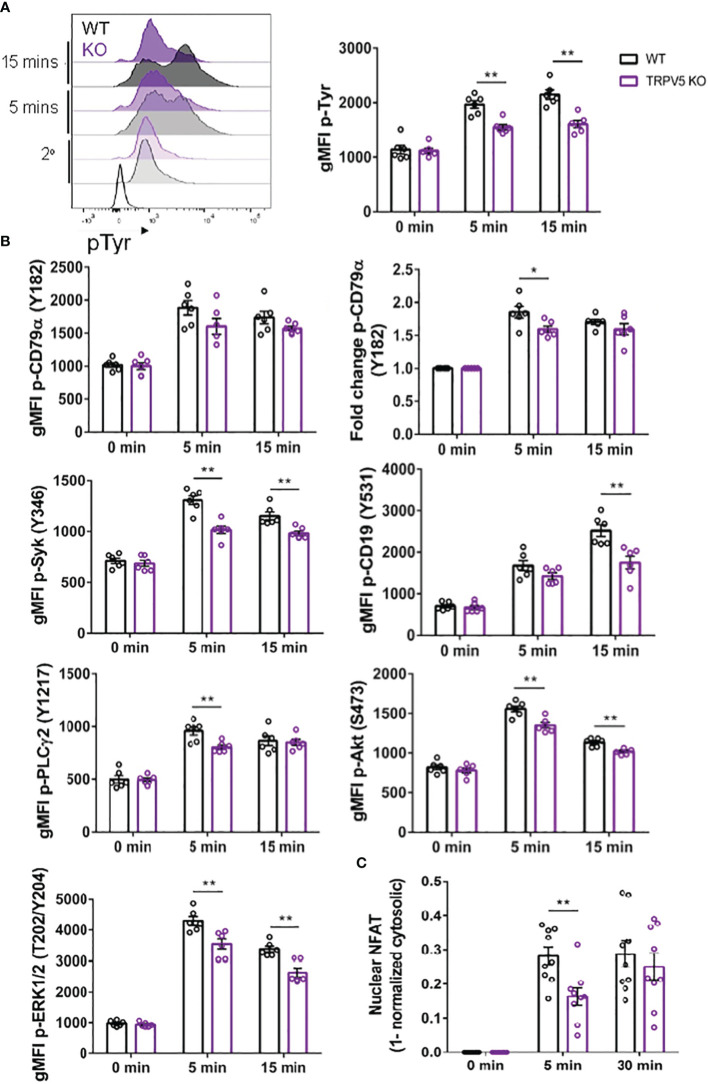
BCR signaling is compromised in TRPV5-deficient B cells. Splenocytes from WT (black) and TRPV5 KO (purple) mice were stimulated for various timepoints with 5 µg/ml of anti-IgM F(ab′)_2_. Cells were either immunostained with anti-B220 and anti-phospho antibodies specific to **(A)** tyrosine residues; **(B)** CD79α, Syk, CD19, AKT, PLC2, or ERK; or **(C)** anti-B220 and anti-NFAT. Cells were then immunostained with **(A)** streptavidin or **(B, C)** secondary antibody. Cells were analyzed using flow cytometry and the **(A, B)** gMFI of phosphorylated proteins, or fold change was determined through **(B)** normalization of gMFI to unstimulated cells or **(C)** normalization of cytosolic levels to unstimulated cells and subtracting this value from 1 to infer nuclear levels were plotted. Data are representative of the mean of six **(A, B)** or nine **(C)** biological replicates, and error bars represent standard error. Statistical analysis was performed using the Mann–Whitney test; *p = 0.026 (pCD79α 5 min), **p = 0.0087 (pAKT 5 min, PLC2 5 min), 0.0022 (pAKT 15 min, pCD19 15 min, pERK 15 min, pSyk 5 min), 0.0043 (pERK 5 min, pSyk 15 min), 0.0056 (NFAT 5 min).

As TRPV5 is necessary for B-cell spreading and BCR microcluster formation, which are the signaling unit in membrane-bound antigen activation, we wanted to determine if TRPV5 is important to BCR signal transduction. To investigate this, we performed phosphoflow analysis of various BCR signaling molecules at 5 and 15 min post-stimulation, timepoints consistent with other studies that have examined BCR signaling (46–48). We found that TRPV5 KO B cells had a significant reduction in global phosphorylation of tyrosine residues upon BCR stimulation (**Figure 7A**). We found that the average gMFI of CD79α (Igα) phosphorylation at Y182 was not significantly decreased at either 5 or 15-min timepoints in TRPV5 KO B cells (**Figure 7B**; **Supplementary Figure S4**); however, we did observe varied basal pCD79α in comparison to other signaling molecules examined. We normalized this variability with a fold change comparison, which revealed that phosphorylation of CD79α at 5 min post-stimulation is significantly decreased in TRPV5 KO B cells (**Figure 7B**; **Supplementary Figure S4**). Loss of TRPV5 also significantly impaired phosphorylation of the early kinase Syk at 5 and 15 min post-stimulation (**Figure 7B**; **Supplementary Figure S4**). We found that early phosphorylation of CD19 at Y531 at 5 min was not significantly decreased in TRPV5 KO B cells; however, there is a trend suggesting a slight reduction (**Figure 7B**; **Supplementary Figure S4**). In contrast, phosphorylation of CD19 at 15 min post-stimulation was significantly reduced in TRPV5 KO B cells (**Figure 7B**; **Supplementary Figure S4**). Phosphorylation of PLCγ2 in TRPV5 KO B cells was significantly reduced at 5 min post-stimulation (**Figure 7B**; **Supplementary Figure S4**), whereas AKT phosphorylation was significantly reduced in TRPV5 KO B cells at both 5 and 15 min post-stimulation (**Figure 7B**; **Supplementary Figure S4**). ERK phosphorylation was also significantly reduced in TRPV5 KO B cells at both 5 and 15 min post-stimulation (**Figure 7B**; **Supplementary Figure S4**).

Activation of calcineurin through calcium influx leads to the activation and dephosphorylation of NFAT, which then translocates to the nucleus (49). As we observed perturbed signal transduction in TRPV5 KO B cells, we wanted to determine whether this had an impact on NFAT translocation upon stimulation. To measure NFAT translocation, we stimulated WT and TRPV5 KO B cells and permeabilized cells after fixation to stain for cytoplasmic NFAT, as previously done in T cells (50). Permeabilization with a low concentration of saponin allowed us to selectively disrupt the plasma membrane and not intracellular membranes (51, 52). We normalized the cytoplasmic NFAT levels to unstimulated cells and subtracted this value from 1 to determine nuclear NFAT levels. We found that TRPV5 KO B cells have a delay in NFAT translocation, as we observed a significant reduction in NFAT translocation into the nucleus at 5 min post-stimulation, but no defect at 30 min post-stimulation (**Figure 7C**). Taken together, these data demonstrated that TRPV5 is important for BCR signal transduction.

### TRPV5 is important for TD antigen-specific IgG responses

**Figure 8:**
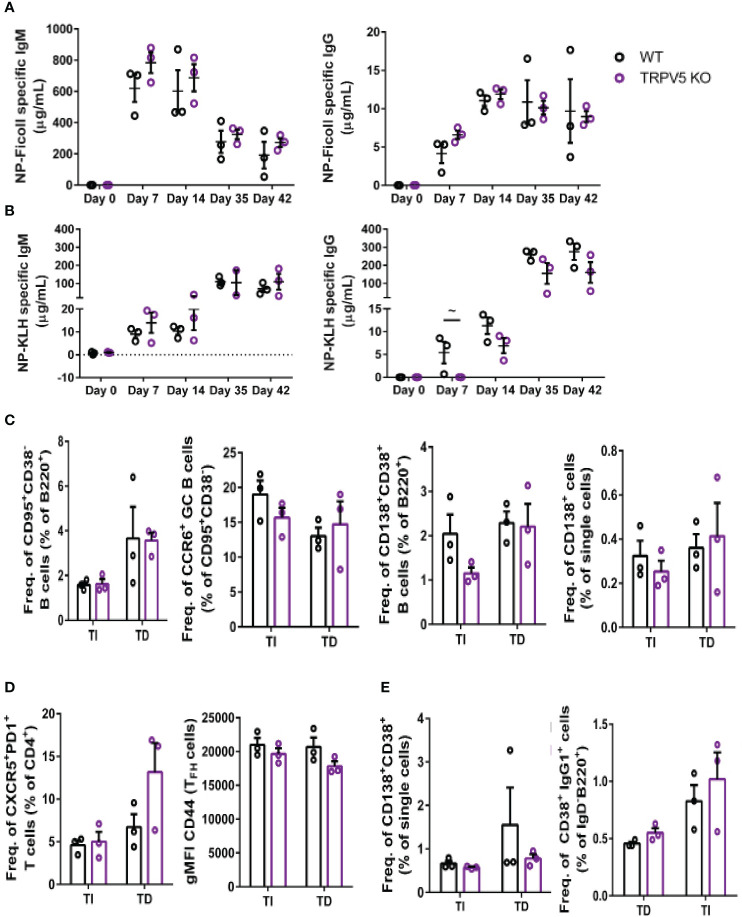
TRPV5 is important for TD antigen-specific IgG production. WT and TRPV5 KO mice immunized with **(A)** DNP-Ficoll or **(B)** DNP-KLH and were bled at days 0, 7, and 14, boosted on day 28, and bled again at days 35 and 42. ELISAs were performed to determine antigen-specific IgM and IgG titers. **(C, D)** Splenocytes or **(E)** bone marrow cells were collected on day 42 (day 14 after boost) from NP-Ficoll- and NP-KLH-immunized WT and TRPV5 KO mice. Cells were immunostained with **(C)** anti-GL7, CD95, CD38, CD138, CCR6, and B220; **(D)** CD4, CD44, PD1, and CXCR5; or **(E)** CD38, CD80, IgD, IgG1, and B220 and were analyzed using flow cytometry. Data are representative of three biological replicates. Graphs for quantification of splenocytes **(C)** gated on B220^+^ to quantify CD38^−^CD95^+^ GC B cells and CCR6^+^ GC B cells, gated on **(B)** B220^+^ or **(E)** single cells to quantify CD38^+^CD138^+^ antibody-secreting cells (ASCs), **(C)** gated on CD4^+^ to quantify PD1^+^CXCR5^+^ TFH cells and CD44 gMFI in TFH cells and **(E)** gated on B220^+^IgD^−^ to quantify IgG1^+^CD38^+^ memory B cells. Statistical analysis was performed using the Mann–Whitney test; n = 3.

We reasoned that the contribution of TRPV5 to BCR signaling *in vitro* may lead to defects in the response of TRPV5-deficient mice *in vivo*. Notably, defects in calcium signaling and calcineurin and NFAT activation have been shown to impact antigen-specific B-cell responses *in vivo* (5, 53–55). We investigated the role of TRPV5 in *in vivo* B-cell responses by immunizing WT and TRPV5 KO mice with either T-independent antigen (TI), DNP-Ficoll, or T-dependent antigen (TD), DNP-KLH. We monitored antibody titers over a primary immunization response for 14 days, boosted at 28 days, and monitored another 14 days before collecting tissue to examine activated cell populations. We found that TRPV5 KO mice had comparable antigen-specific IgM and IgG responses to TI antigen immunization (**Figure 8A**). We also found that TRPV5 KO mice have comparable TD antigen-specific IgM titers to KLH immunization (**Figure 8B**). However, we found that TRPV5 KO mice have an impairment in TD antigen-specific IgG titers after KLH immunization at day 7, and this trend (p = 0.06, denoted by ~) continues throughout the immunization timeline (**Figure 8B**). Thus, our data suggest that TRPV5 is important for the production of early TD antigen-specific IgG upon immunization.

In addition to assessing antibody production, we characterized the cellular immune response of immunized mice on day 42 using flow cytometry. We found that GC B cells and memory B-cell precursors were comparable in WT and TRPV5 KO mice immunized with either Ficoll or KLH (**Figure 8C**). In contrast, we found a non-significant decrease in plasmablasts in the spleens of TRPV5 KO mice immunized with TI antigen (**Figure 8C**). This was not seen in TD antigen-immunized TRPV5 KO mice (**Figure 8C**). However, antigen-specific antibody titers were not impaired suggesting that this decrease does not impact TI antigen responses in TRPV5 KO mice. We also observed a non-significant increase in T_FH_ cells in KLH-immunized TRPV5 KO mice compared to WT, which was not observed in Ficoll-immunized mice (**Figure 8D**). The activation level of T_FH_ cells in TRPV5 KO mice was also observed to trend slightly lower (not statistically significant) when measured by CD44 expression (**Figure 8D**). Additionally, we stained for plasma cells (CD38^+^CD138^+^) and memory B cells (B220^+^IgD^-^CD38^+^IgG1^+^) in the bone marrow. We found that plasma cell and memory B cell numbers in the bone marrow were comparable between WT and TRPV5 KO mice immunized with either Ficoll or KLH (**Figure 8E**). The decreased plasmablast levels in the spleens of Ficoll-immunized TRPV5 KO mice do not seem to impact the plasma cell population in the bone marrow (**Figure 8C**). Overall, these findings suggest that TRPV5 is important for TD antigen-specific antibody responses *in vivo*, and perturbed T_FH_ cell numbers and activation status may contribute to this observation. Additionally, despite lower plasmablast numbers of TI antigen-immunized TRPV5 KO mice, TRPV5 does not impact TI responses *in vivo*.

## Discussion

Here, we demonstrate that TRPV5 is a positive regulator of B-cell activation. We found that TRPV5 is expressed in primary WT B cells and polarizes to BCR clusters during soluble and membrane-bound antigen stimulation. This polarized fraction contains membrane-localized TRPV5 that localizes with BCR microclusters in a BCR signaling-dependent manner. This finding is in agreement with previous findings that TRPV5 activity is regulated by trafficking and insertion into the plasma membrane upon various stimuli (25–27, 39–43, 56–58). In contrast to these studies, however, we found that intracellular and membrane TRPV5 levels are not altered upon BCR stimulation. Instead, membrane-localized TRPV5 is recruited to BCR clusters in primary B cells. We do see co-localization of TRPV5 and IgM even at later timepoints of stimulations suggesting the internalization of TRPV5 and IgM into endocytic compartments. This correlated with a decrease in membrane-localized TRPV5 at 30 min post-stimulation. Inhibition of PI3K, RhoA, and Myosin IIa all impaired TRPV5 polarization upon BCR stimulation demonstrating a BCR signaling intrinsic recruitment of TRPV5 to the BCR cap. Surprisingly, inhibition of Src and Syk kinases did not impair TRPV5 polarization despite their activation being upstream of the PI3K pathway. Inhibition of class I PI3Ks by pictilisib impairs PIP3 generation and protein translocation from vesicles to the plasma membrane (59, 60). PI3K is also required for BTK and PLCγ2 activation in stimulated B cells, and perhaps, the activity of one of these proteins is required for TRPV5 polarization (61–63). Our studies do not rule out the possibility of vesicle-localized TRPV5 coming in close proximity to BCR clusters on the plasma membrane of B cells without being inserted into the membrane. However, the requirement for RhoA activity for TRPV5 localization does suggest the need for PIP2 synthesis in B cells for the polarization of TRPV5 (35) and may be involved in regulating the activity of TRPV5 in B cells (36, 37).

We demonstrate that TRPV5 does not contribute to global calcium signaling upon BCR stimulation. This is surprising as TRPV5 is calcium selective and predominantly shuttles calcium when this ion is present in media (24). A close relative, TRPV6, is expressed in CD4^+^ T cells; however, genetic deletion of this ion channel did not impact calcium signaling in T cells upon TCR stimulation (3). Taken together, these findings suggest that these calcium-selective TRPs may play alternative roles in immune cells. However, we found that TRPV5 KO B cells have a different transcriptional profile with upregulated expression of multiple calcium channels and regulatory proteins. These changes in mRNA expression in TRPV5 KO B cells suggest that these cells need to compensate for the loss of calcium influx activity from TRPV5. Thus, despite no statistically significant differences in calcium signaling, our data suggest that TRPV5 is likely important for calcium signaling in B cells. Similar findings were shown in TRPV2 KO B cells where STIM1, ORAI1, and ORAI2 transcription levels were slightly increased and potentially compensated for calcium signaling when TRPV2 is not present (13). Our finding of significantly increased SERCA3 and increased TRPM7 expression in TRPV5 KO B cells suggests that deficiency of TRPV5 disrupts calcium shuttling into the ER implying that perhaps TRPV5 plays an unidentified role in intracellular calcium regulation in B cells.

Despite the lack of a calcium signaling defect in TRPV5 KO B cells, we found that TRPV5 is important for B-cell spreading and contraction upon interaction with a membrane-bound antigen. Previous work has demonstrated that B cells deficient in key BCR signaling molecules, e.g., Lyn, Syk, BTK, PLCγ2, and CD19, have impaired spreading and contraction responses (34, 64). Inhibiting IP_3_Rs in mouse A20 B cells also impaired cell adhesion and spreading in response to membrane-bound antigen (65). The related channel, TRPV2, has also been found to be present in the immunological synapse, and knocking down this channel results in impaired synapse formation and B-cell signaling (13). The defect in spreading and antigen aggregation observed in TRPV5 KO B cells demonstrates that TRPV5 plays an important role in B-cell interactions with membrane-bound antigen and signalosome formation. TRPV5 KO B cells also have a notable impairment in signal transduction upon BCR stimulation, as phosphorylation of tyrosine residues on several key signaling proteins is reduced. We also observed an early defect in NFAT activation, as NFAT translocation to the nucleus is delayed in TRPV5 KO B cells. These findings clearly demonstrate an important role for TRPV5 in BCR signaling and B-cell activation, although the molecular mechanism for altered BCR signaling in TRPV5-deficient B cells requires further study. TRPV2 was recently shown to contribute to cytoskeleton remodeling, immunological synapse formation, and B-cell activation through mediating cation signaling and membrane potential depolarization (13). While TRPV5 is permeable to other cations while being calcium selective, it is also possible that localized TRPV5-mediated calcium influx in the vicinity of BCR microclusters is altered in TRPV5-deficient cells, despite no statistically significant defect in global calcium signals measured using flow cytometry.

Finally, we see that TRPV5 is important for early TD antigen-specific IgG production upon immunization. Extracellular plasmablast (EXPB) production is what leads to the first wave of antibody production before the initiation of germinal centers and the differentiation of B cells, either as plasma cells or memory B cells to contribute to the immune response. IgG^+^ EXPBs have been shown to rely on strong BCR signaling and T cell help after the B cell first encounters an antigen and gets activated (66–69). Our data suggest that the impairment in BCR signaling in TRPV5 KO B cells may be below the threshold required for IgG^+^ EXPB formation. Alternatively, TRPV5 KO B cells may have an impairment in antigen presentation to obtain T-cell help resulting in a loss of antigen-specific antibodies produced by IgG^+^ EXPBs. This warrants future work to determine the role of TRPV5 in TD antigen-specific IgG titers.

Our study is the first, to our knowledge, to demonstrate TRPV5 expression in B cells and investigate the role of this ion channel in B-cell development, signaling, activation, and effector function. Our study identifies an important role for TRPV5 in B-cell activation *in vitro* and *in vivo* emphasizing the importance of investigating novel ion channels expressed in B cells. Studying alternative calcium channels, such as TRPV5, in B-cell activation and signaling will help us understand the role of ion channels in the immune response while also shedding light on calcium signaling in B cells. Discovery and investigation of alternative non-SOCE calcium channels will also help resolve the surprising observation that knocking out components of the SOCE pathway in B cells does not impact humoral immunity. Investigating ion signaling in B cells may also help develop potential therapeutics that target ion channels in the treatment of B cell-mediated diseases.

## Data availability statement

The raw data supporting the conclusions of this article will be made available by the authors, without undue reservation.

## Ethics statement

The animal study was approved by Local Animal Care Committee (LACC) at the University of Toronto. The study was conducted in accordance with the local legislation and institutional requirements.

## Author contributions

TM: Conceptualization, Writing – original draft, Writing – review & editing, Data curation, Formal analysis, Investigation, Methodology. HS: Formal analysis, Investigation, Writing – review & editing. LS: Formal analysis, Investigation, Writing – review & editing. LB: Formal analysis, Investigation, Writing – review & editing. AB: Formal analysis, Investigation, Writing – review & editing. NG: Supervision, Writing – review & editing. BT: Conceptualization, Funding acquisition, Project administration, Resources, Supervision, Writing – original draft, Writing – review & editing.
