# Supplementary material for: The ion channel TRPV5 regulates B-cell signaling and activation
Source: Front Immunol. 2024 Apr 17;15:1386719. doi: 10.3389/fimmu.2024.1386719 (PMC11061418; doi:10.3389/fimmu.2024.1386719)
Supplement: Supplementary file 1 [file DataSheet_1.pdf]

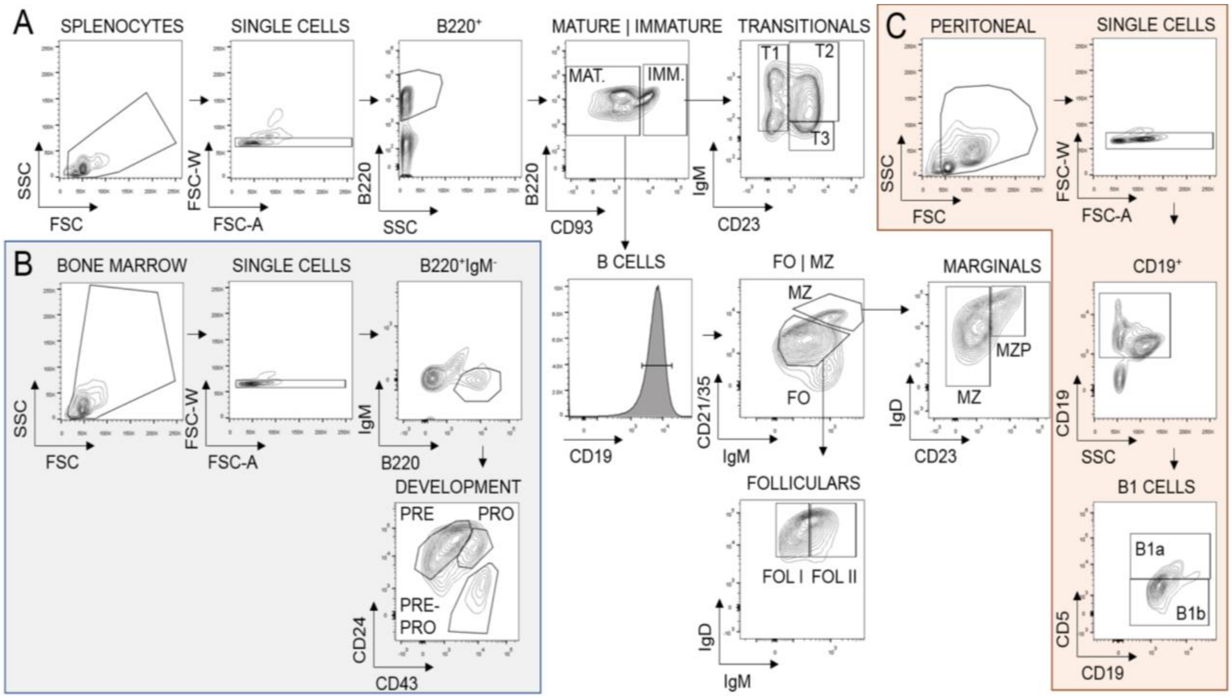

Figure S1. Determining TRPV5 expression in B cell subsets

Gating strategy to identify various B cell subsets from (A) splenocytes, (B) bone marrow cells and (C) peritoneal cells.

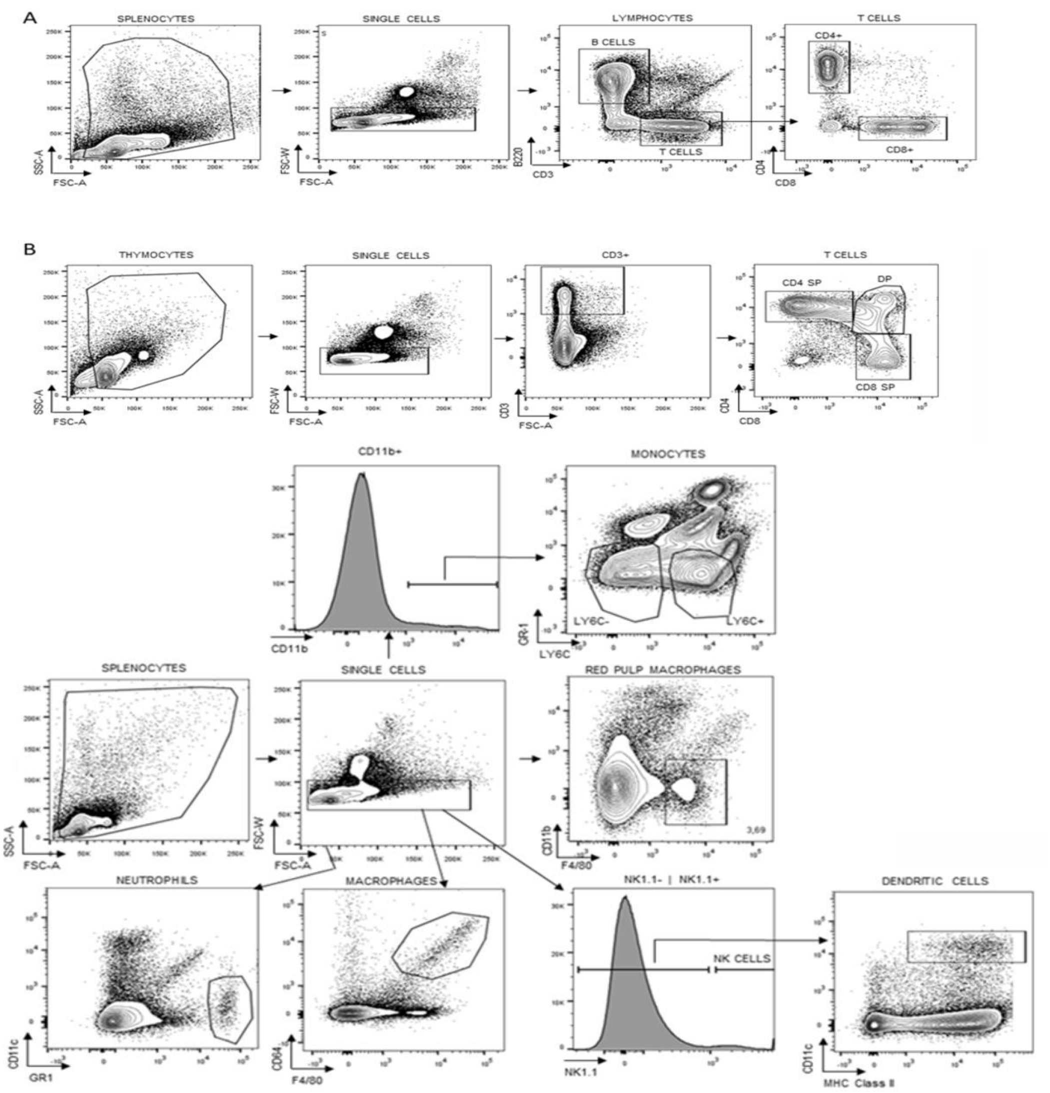

Figure S2. Investigating immune cell compartments in TRPV5 KO mice
(A) Gating strategy to identify T cell subsets from splenocytes. (B) Gating strategy to identify T cell subsets from thymocytes. (C) Gating strategy to identify splenic innate immune cell subsets.

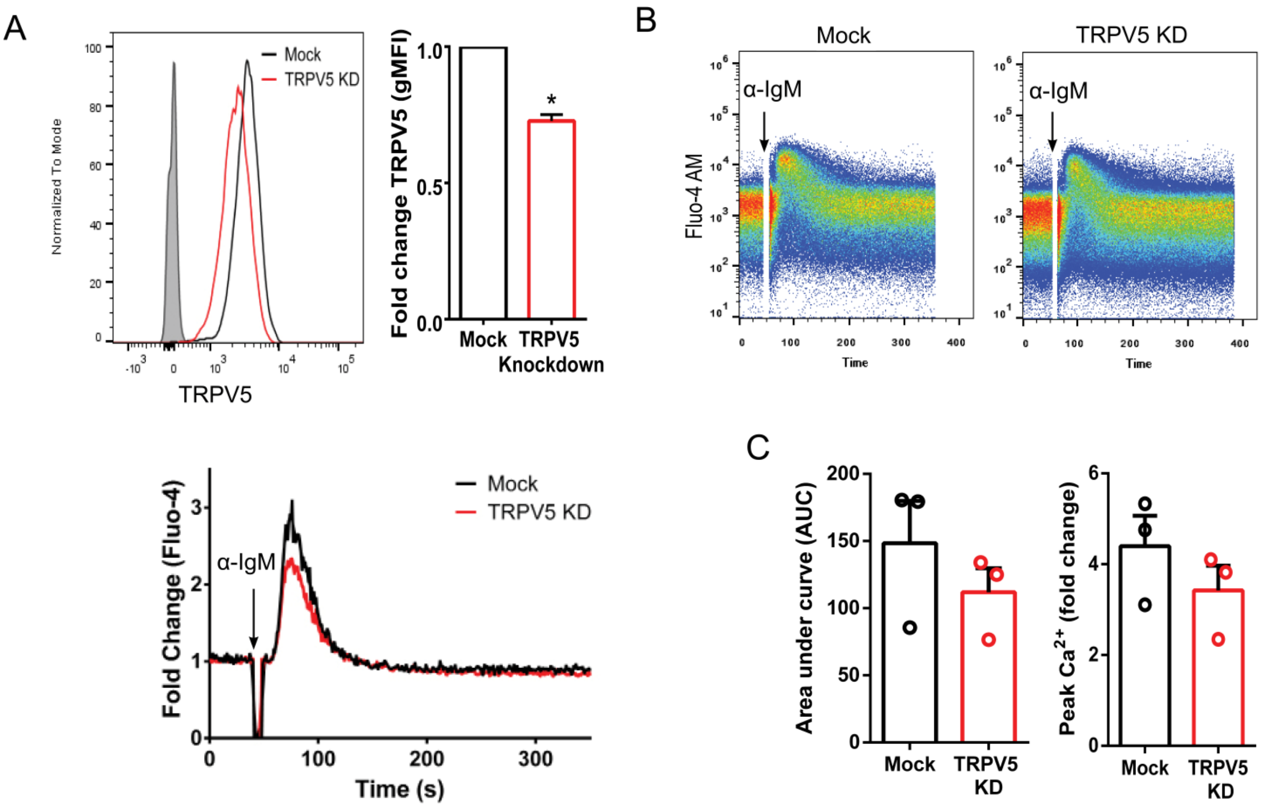

Figure S3. Modest siRNA knockdown of TRPV5 in a murine B cell line reduces calcium signalling upon BCR stimulation.

(A) D1.3 A20 B cells transfected with TRPV5 siRNA were immunostained for TRPV5 expression 48 hours post-transfection. Mock (black), siRNA knockdown (red) and unstained (grey) cells were fixed and immunostained for TRPV5 (4ADI). These cells were analyzed by flow cytometry. n=3. (B) Cells were labeled 48 hours post-transfection with the calcium indicator fluo-4 and calcium flux upon BCR stimulation was monitored by flow cytometry. Black arrow indicates addition of stimulating antibody. Fold change of Fluo-4 signal over time in mock (black line) and siRNA-treated (red line) cells, n=3. (C) Area under the curve (AUC) and peak Ca2+ levels were calculated and graphed from the calcium flux plots on GraphPad Prism, n=3.

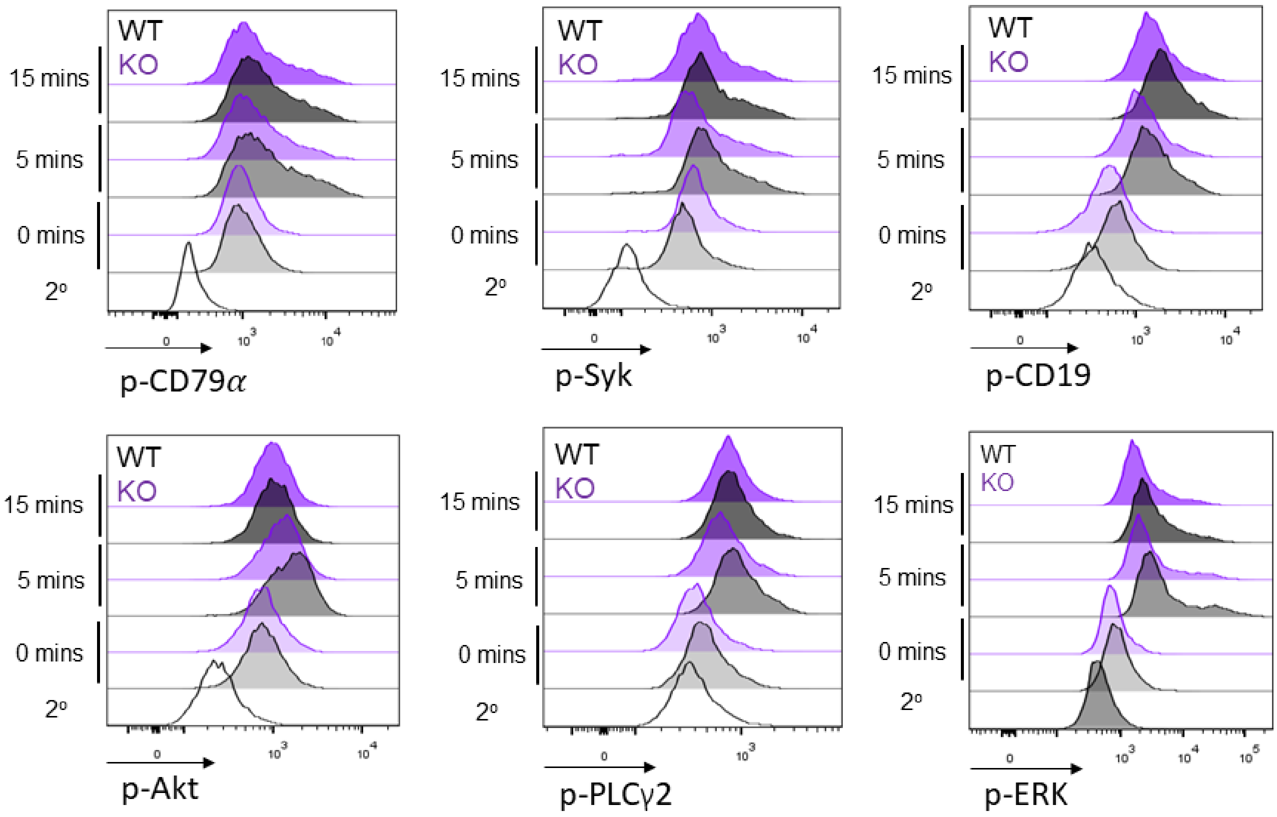

Figure S4. Representative flow plots for signalling molecules after BCR stimulation. Splenocytes from WT (black) and TRPV5 KO (purple) mice were stimulated for various time-points with 5 µg/mL anti-IgM F(ab')$_2$. Cells were either immunostained with anti-B220 and anti-phospho antibodies specific to CD79α, Syk, CD19, AKT, PLCγ2, or ERK. Cells were then immunostained with secondary antibody. Cells were analyzed by flow cytometry.
